# Tumor Targeting by Monoclonal Antibody Functionalized Magnetic Nanoparticles

**DOI:** 10.3390/nano9111575

**Published:** 2019-11-06

**Authors:** Francesca Oltolina, Donato Colangelo, Ivana Miletto, Nausicaa Clemente, Marta Miola, Enrica Verné, Maria Prat, Antonia Follenzi

**Affiliations:** 1 Laboratory of Histology, Department of Health Sciences (DSS), Università del Piemonte Orientale “A. Avogadro”, Via Solaroli 17, 28100 Novara, Italy; 2 Laboratory of Pharmacology, Department of Health Sciences (DSS), Università del Piemonte Orientale “A. Avogadro”, Via Solaroli 17, 28100 Novara, Italy; 3 Department of Science and Technological Innovation (DISIT), Università del Piemonte Orientale “A. Avogadro”, Viale Teresa Michel 11, 15100 Alessandria, Italy; 4 Laboratory of Immunology, Department of Health Sciences (DSS), Università del Piemonte Orientale “A. Avogadro”, Via Solaroli 17, 28100 Novara, Italy; 5 Department of Applied Science and Technology (DISAT), Politecnico di Torino, Corso Duca degli Abruzzi 24, 10129 Torino, Italy; 6 Centro di Biotecnologie per la Ricerca Medica Applicata (BRMA), Via Solaroli 17, 28100 Novara, Italy; 7 Consorzio Interuniversitario per Biotecnologie (CIB), Località Padriciano 99, 34149 Area di Ricerca, Italy; 8 Consorzio Interuniversitario Nazionale per la Scienza e Tecnologia dei Materiali (INSTM), 28100 Novara, Italy

**Keywords:** magnetic nanoparticles, tumor targeting, monoclonal antibodies, cytotoxicity, doxorubicin

## Abstract

Tumor-targeted drug-loaded nanocarriers represent innovative and attractive tools for cancer therapy. Several magnetic nanoparticles (MNPs) were analyzed as potential tumor-targeted drug-loaded nanocarriers after functionalization with anti-Met oncogene (anti-Met/HGFR) monoclonal antibody (mAb) and doxorubicin (DOXO). Their cytocompatibility, stability, immunocompetence (immunoprecipitation), and their interactions with cancer cells in vitro (Perl’s staining, confocal microscopy, cytotoxic assays: MTT, real time toxicity) and with tumors in vivo (Perl’s staining) were evaluated. The simplest silica- and calcium-free mAb-loaded MNPs were the most cytocompatible, the most stable, and showed the best immunocompetence and specificity. These mAb-functionalized MNPs specifically interacted with the surface of Met/HGFR-positive cells, and not with Met/HGFR-negative cells; they were not internalized, but they discharged in the targeted cells DOXO, which reached the nucleus, exerting cytotoxicity. The presence of mAbs on DOXO-MNPs significantly increased their cytotoxicity on Met/HGFR-positive cells, while no such effect was detectable on Met/HGFR-negative cells. Bare MNPs were biocompatible in vivo; mAb presence on MNPs induced a better dispersion within the tumor mass when injected in situ in Met/HGFR-positive xenotumors in NOD/SCID-γ^null^ mice. These MNPs may represent a new and promising carrier for in vivo targeted drug delivery, in which applied gradient and alternating magnetic fields can enhance targeting and induce hyperthermia respectively.

## 1. Introduction

Despite the substantial advancements both in diagnosis and therapy, cancer is still the second cause of morbidity and mortality in western countries, although great advances have been observed both in diagnosis and therapy [1]. Chemotherapy, one of the most important conventional cancer therapies, suffers from several limitations, such as lack of specificity and drug-associated side effects, which can be particularly severe as the well-known cardiotoxicity of anthracyclines [2,3,4]. In the field of nanotechnology, nanoparticles (NPs), in particular, which may overcome these drawbacks are actively studied and applications have started to be found [5]. Indeed, thanks to their nanometric dimensions, and thus their high surface to volume ratio, nanocomplexes can be very efficient multifunctional platforms for what is called presently theranostics, acting not only as drug delivery systems, but also for tumor imaging, and tracking with fluorophore and other molecules to follow the disease and its treatment [6,7,8,9,10,11]. Moreover, NPs can increase drugs bioavailability and protect them from degradation [12]. More importantly, they can exert tumor targeting activity, both passive and active, minimizing the drug-associated adverse side effects and increasing the therapeutic index [6,13].

Concerning active targeting, magnetic nanoparticles (MNPs) present the inherent physical properties of being able to respond to an applied magnetic field, which can direct them on the targeted tumor site, where they can release their payload [14]. MNPs can also respond to an alternating magnetic field, developing hyperthermia, which can be exploited for thermoablation of cancer cells [15,16,17]. Moreover, MNPs can be used in magnetic resonance imaging (e.g., as contrast agents), cell transfections, and also for in vivo cell tracking and tissue repair [10,18,19].

MNPs are generally obtained by chemical synthesis, more often by coprecipitation of Fe^2+^ and Fe^3+^ ions in a basic aqueous media (e.g., NaOH or NH_4_OH solutions). The small magnetic core based on iron oxide (Fe_3_O_4_ herein named M) can be coated with a shell, with the goal of stabilizing the MNP, avoiding their aggregation and adding functionalities [11,20,21]. MNPs smaller than 30 nm generally display superparamagnetic properties [12].

Active tumor targeting can be pursued also by engrafting drug-loaded NPs with probes recognizing specifically tumor-associated markers expressed at the cell surface. This is the evolution and the improvement of the immunotoxins and antibody-drug conjugates, which first realized the concept of “magic bullet” [22,23], since NPs can carry significantly higher amounts of both targeting moieties and chemotherapeutics, and thus are more efficient, displaying higher avidity for the targeted tumor cells [24,25,26].

Receptors for growth factors are among the most used targetable tumor-associated markers, as they are often overexpressed on tumor cells, since their overexpression maybe pivotal and responsible for the uncontrolled proliferation of tumor cells. Well-known examples are the folate receptor [27], the transferrin receptor [28], the tyrosine kinase receptors, such as for epidermal growth factor HER2, and hepatocyte growth factor [29,30,31,32]. The latter is encoded by the Met oncogene (Met/HGFR) and is overexpressed in many tumors and involved also in the metastatic process [31,32,33]. Different types of targeting ligands can be used; monoclonal antibodies (mAbs) or their fragments and growth factors are among the most common [24,27,28], but also peptides [34,35] and aptamers [36,37] are used.

**Figure 1:**
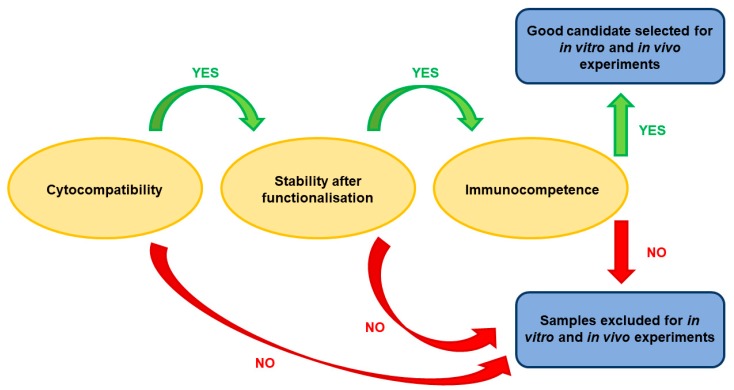
Strategy for the selection of the best nanoparticle for in vitro and in vivo experiments.

In this study, different types of MNPs (uncoated or coated with silica, undoped or doped with calcium) were studied with the aim of selecting the best one for in vitro and in vivo experiments after functionalization with monoclonal antibodies against the Met/HGFR as targeting moieties and with doxorubicin (DOX0), a commonly used chemotherapeutic drug. Different properties and parameters were taken into account for selection at different subsequent steps, as shown in Figure 1. We show that uncoated and undoped MNPs are the best performing. Moreover, mAb-functionalized MNPs specifically interact with the surface of Met/HGFR-positive GTL-16 cells, and not with HGF-R/Met-negative Huh7 cells. The presence of the mAbs on DOXO-MNPs (mAb-DOXO-MNPs) significantly increased the toxic activity on GTL-16 cells, whereas no such effect was detectable on Huh7 cells. MNPs were biocompatible when injected in mice and the presence of the mAb on MNPs enhanced their retention (in the tumor) when injected in situ in GTL-16 cells induced xenograft tumors in γ-null mice.

## 2. Materials and Methods

### 2.1. Materials

Unless otherwise specified reagents were purchased from Sigma-Aldrich, St. Louis, MO, USA. DMEM high glucose (L-glutamine 4 mM, glucose 4500 mg L^−1^) and phosphate buffer saline (0.0067 M, none of calcium and magnesium), fetal calf serum (FCS), annexin V-FITC and horseradish peroxidase-conjugated affinity purified rabbit anti-mouse IgG antibodies were purchased from Thermo Fisher (Waltham, MA, USA). Enhanced chemiluminescence (ECL kit) from Biorad (Hercules, CA, USA); and polyvinyl fluoride membranes for western blot from GE Healthcare Life Science (Chicago, IL, USA). TRITC-phalloidin, FITC-labelled rabbit anti-mouse Ig and TO-PRO-3 were purchased from Life Technologies (Carlsbad, CA, USA). All reagents were of analytical purity and used without further purification.

### 2.2. Synthesis of Superparamagnetic Iron-Oxide Nanoparticles

MNPs were synthetized as already described [38,39]. All the MNPs described consisted of a core of iron oxide, obtained by a co-precipitation method mixing aqueous solutions of FeCl_2_·4H_2_O and FeCl_3_·6H_2_O 0.01 M to obtain a stoichiometric ratio of Fe^2+^/Fe^3+^ of 1:2, with the addition of NH_4_OH. Citric acid (0.05 M) was added and adsorption was carried out at 80 °C, before the last washing to improve the MNPs dispersion in water. Coating with Silica and doping with calcium were also carried out in view of a possible improvement of MNP water dispensability and greater potential for their further functionalization [20,38]. In particular, some MNPs were coated with a silica shell (MSNPs) following the Stöber method [40], by the addition of a mixture of tetraethyl orthosilicate (TEOS) as silica precursor, ethanol and water (with an ethanol:water ratio of 1:1). Finally, some MNPs were also prepared, after TEOS functionalization, in the presence of calcium citrate (MSCaC-NP) or calcium hydroxide (MSCaH-NP), reaching a very low Si:Ca ratio (99:1), to avoid particles agglomeration and toxic effect towards cells, but to enhance surface reactivity [41,42].

### 2.3. Functionalization of Nanoparticles

Magnetic nanoparticles were functionalized with DOXO and with the DO-24 monoclonal antibody (mAb), an IgG2a/k mAb produced against the ectodomain of the human Met/HGF receptor [43,44] by isothermal adsorption, as already described [45]. Experiments were performed to assess the maximum amount of mAb and/or DOXO loaded on the nanoparticles separately and in combination by mixing 5 mg of MNPs with 1 mg/mL of DOXO dissolved in water or of purified mAb dissolved in Hepes buffered saline solution (0.01 M Hepes, 0.15 M NaCl). Mixtures were shaken at 200 rpm at 25 °C for different periods of time up to 24 h in Eppendorf tubes. DOXO and mAb contents were assessed by UV-Vis spectroscopy (λ = 495 and 280 nm, ε = 10,650, and 64,000 M-1 cm^−1^, respectively; Nanodrop 2000, ThermoScientific). The amounts of the adsorbed molecules were calculated from the differences between the concentrations of the molecules in solutions before and after the adsorption on MNPs (the so-called supernatants). The solid components were washed many times with Hepes buffered saline solution until the absorbance was less than 0.02 absorbance arbitrary units at 490 or at 280 nm (equivalent to a negligible amount) using a magnet to avoid removing the MNPs. Then, the functionalized nanoparticles were resuspended in Hepes buffered saline solution and kept at 4 °C until their use. In the case of MNPs conjugated with both moieties (mAb and DOXO, the so-called ternary nanoparticles), the same protocol was sequentially applied, each time followed by extensive washings, DO-24 mAb coupling first and DOXO coupling in the second step, based on previous experiments of the group [9,45].

### 2.4. Characterization of Nanoparticles

The stability of the functionalized MNPs was monitored by incubating them in phosphate buffered saline (PBS) containing 10% fetal calf serum (FCS) or not for different periods of time (from 1 to 7 days, two samples for each time) under constant stirring (200 rpm) at 37 °C. In the case of DOXO-functionalized NPs, supernatants were, then, separated from the solid components by a magnet and analyzed by UV-Vis spectroscopy, as described above and the released amounts were referred to as a percentage of the amounts that were adsorbed initially. The stability of the mAb-functionalized nanoparticles was assessed directly on the NPs recovered by the magnet and the mAb bound on them was estimated in SDS-PAGE and WB.

The size distribution of MNPs was evaluated by dynamic light scattering (DLS) measurements, using a Zetasizer Nano-ZS instrument (Malvern Instruments, Worcestershire, UK) equipped with a 4 mW He-Ne laser operating at 633 nm and a detection angle of 173°. For the measurements, the materials were suspended (0.1 mg/mL) in PBS (pH 7.4). Hydrodynamic diameters are reported as number distribution and expressed as mean value ± standard deviation of three separate measurements, each resulting from 14 runs.

The ζ potential of the MNPs was measured by electrophoretic light scattering (ELS), using a ZetasizerNano-ZS (Malvern Instruments). For the measurements, the materials were suspended in MilliQ water at the concentration 0.1 mg and for each sample five measurements were carried out, each resulting from 10 runs. The ζ potential values are, therefore, expressed as mean value ± standard deviation.

The immunocompetence of the mAb-functionalized MNPs was assessed by incubating them with clarified detergent extracts prepared from GTL-16 (Met+) cells and Huh7 (Met–), as negative controls, in DIM buffer (50 mM PIPES pH 7.4, 300 mM saccharose, 100 mM NaCl, 5 mM EGTA, 5 mM MgCl_2_, and 100 μM ZnCl_2_), 1% Triton X-100, 1 mM TRIS HCl pH 8.8, and a cocktail of protease inhibitors) at 4 °C overnight, as described [45]. MNPs were then washed three times by the use of a magnet, proteins were solubilized, heated at 95 °C, separated in 10% SDS-PAGE, transferred on polyvinyl fluoride membranes for Western blot and decorated with anti-Met DL-21 mAb [44]. Then filters were reacted with horseradish peroxidase-conjugated affinity purified rabbit anti-mouse Ig antibodies (RaMIg/PO, 1:5000), reacted with enhanced chemiluminescence (ECL) and analyzed in the Versadoc instrument (Biorad, Hercules, CA, USA).

Micro-Raman analyses were carried out using a Jobin Yvon HR800 LabRam spectrometer (HORIBA Jobin Yvon, Paris, France). Instrument calibration was carried out before each analysis by checking the position and intensity of the Si band at 520.65 + −0.05 cm^−1^. In order to balance signal against noise, at least two cycles of 300 s were performed. A D06 filter allowed preventing damages to the samples and the confocal hole was maintained at 200 µm to collect signal from the surface of the particles. All the spectra were collected with an 80× objective, with a 0.75 numerical aperture (N/A), and 1800 gr/mm diffraction grating. All the physical parameters were fixed during every analysis. Spectral resolution of the instrument was about 2 cm^−1^ and the registered spectra were processed by means of the ORIGIN software v. 6.0 (OriginLab Corporation, Northampton, MA, USA).

### 2.5. Cell Lines

GTL-16 [46], a poorly differentiated human gastric carcinoma derived cell line which express Met/HGFR, and Huh7 [47], a well differentiated hepatocyte derived cell carcinoma negative for Met/HGFR expression, were maintained in Dulbecco’s modified Eagle’s medium (DMEM, Sigma), supplemented with 10% FCS, 50 U/mL penicillin, and 50 μg/mL streptomycin. Cells were incubated at standard conditions (37 °C, 5% CO2), and passaged when they were at 90% to 95% confluence (at ratios, GTL-16 of 1:3 and Huh7 of 1:6).

### 2.6. Cytocompatibility

For cytocompatibility assays GTL-16 cells (12 × 10^3^/well) and Huh7 (6 × 10^3^/well) were incubated in 100 μL of complete medium, and then, several concentrations of the different nanoparticles or mAb-functionalized nanoparticles were added in 100 μL of fresh medium [45]. Doxorubicin (DOXO), 1 μg/mL, was used as control of toxicity. After 3 days incubation, cell viability was evaluated by the 3-(4,5-dimethylthiazol-2-yl)-2,5-diphenyltetrazolium bromide (MTT, Sigma) colorimetric assay. Briefly, 20 µL of MTT solution (5 mg/mL in a PBS solution) were added to each well. The plate was then incubated at 37 °C for 3 h. After the removal of the solution, 125 µL of 0.2 M HCl in isopropanol were added to dissolve formazan crystals. One hundred µL were then removed carefully and the optical density was measured using a multi-well plate reader (Multilabel Reader Victor TM X4; PerkinElmer (Waltham, MA, USA) at 570 nm. Viability of parallel cultures of untreated cells was taken as 100% viability, and values obtained from cells undergoing the different treatments were referred to this value. Experiments were performed 4 times using 3 replicates for each sample.

For cytofluorometer assessment of cell death, cells were collected after 72 h incubation with nanoparticles, washed in PBS, and incubated for 15 min at room temperature with 2 µL of annexin V-FITC (100 nM) and 5 µL of propidium iodide (PI, 50 µg/mL) dissolved in 93 µL of buffer solution (10 mM HEPES-NaOH, pH 7.4, 140 mM NaCl, and 2.5 mM CaCl_2_). At least 10,000 cells were analyzed in a FACScan flow cytometer (Becton Dickinson, Mountain View, CA, USA) equipped with an argon laser and the Leica TCS SP2 AOBS Spectral Confocal Scanner microscope. All experiments were performed in triplicate and repeated at least three times. Data are given as means ± SD. Couples of conditions were compared with Student’s *t*-test. The level of significance was *p* < 0.05.

### 2.7. Interaction of Magnetic Nanoparticles with Cells and Cytotoxicity of Functionalized MNPs

For Prussian Blue (PB) staining GTL-16 (24 × 10^3^/well) and Huh-7 (12 × 10^3^/well) were seeded on glass coverslips in 24-well plates and after 24 h presaturated MNPs (in 0.4% BSA at 37 °C for 2 h), either not functionalized or mAb-functionalized, were added (100 µg/mL). After the incubation at 37 °C for 2 h, coverslips were washed with fresh PBS pH 7.2, fixed with paraformaldehyde (4 wt. % in PBS) and rinsed in PBS. Then, they were stained with Prussian Blue solution (1:1 of 2% potassium ferrocyanide and 2% HCl both in H_2_O) for 15 min, as described [38]. After washing with fresh PBS, nuclear fast red was added to stain cell nuclei. Coverslips were then washed with H_2_O and were mounted on slides with Eukitt quick-hardening mounting medium. Samples were analyzed by optical microscopy.

Alternatively, fixed samples were processed for confocal microscopy analysis. For this they were permeabilized with TRIS-buffered saline (TBS) containing 5% bovine serum albumin (BSA, Sigma), 0.1% Triton-X100 and 5% goat serum and then stained for 1 h, as described [45]. Cytoskeletal actin was stained with TRITC-phalloidin (1/200, excitation at 543 nm, and emission at 560–620 nm), or FITC-phalloidin (excitation at 488 nm and emission at 500–535 nm), mAbs with FITC-labelled rabbit anti-mouse IgG (1/100, excitation at 488 nm, and emission at 500–535 nm) and nuclei with TO-PRO-3 (1/70, excitation at 633 nm and emission at 650–750 nm). DOXO was detected after excitation at 476 nm and emission at 575–630 nm. At the end of the incubation, slides were rinsed and mounted. Fluorescence was detected using the Confocal Scanner microscope. Images were taken at 630× magnification.

For cytotoxicity experiments performed with the xCELLigence^®^ instrument (RTCA system; ACEA Biosciences Inc., Santa Clara, CA, USA) cells (approximately 12 × 10^3^ GTL-16/well or 6 × 10^3^ HuH-7/well) were seeded on appropriate micro-well plates for 24 h. From this time on, the impedence was monitored (time 0 of the experiment), and the not functionalized or the differently functionalized MNPs (100 μg/mL) were added in 100 μL of medium. Equimolar amounts of DOXO, either soluble or loaded on nanoparticles were used. Cell cultures were monitored up to 3 days.

### 2.8. In Vivo Distribution of MNPs

All procedures were approved (Ministero della Salute: #178/2019-PR) and carried out in accordance with the Animal Care and Use Committee of UPO, the European Community Directive for Care and Italian Laws on animal experimentation (Law by Decree 116/92). Mice were purchased from Charles River (Calco, Lecco, Italy) and housed under standard conditions in a pathogen-free environment.

Three 8-to-10-week old NOD/SCID-γ^null^ (NSG) female mice were injected in the tail vein with 10 µg/g mouse diluted in a final volume of 100 µL of sterile PBS. After 3 days these mice, together with untreated control mice, were euthanized and their organs were collected, fixed, embedded in paraffin, and processed for histological analysis. Serial sections were stained with Prussian blue, counterstained with nuclear fast red and subjected to histological evaluation by an independent pathologist not informed of the sample identity.

### 2.9. In Vivo Injection of MNPs in Tumor-Bearing Animals

For tumor formation 2 × 10^6^ human GTL-16 cells/mouse were resuspended in PBS, subcutaneously injected and allowed to grow up to 7–10 days (tumor size was around 0.5 × 0.5 cm). Fourteen mice were then subdivided in two groups and injected intratumorally with either not functionalized MNPs or mAb-functionalized MNPs (10 µg MNP/g mouse). A third group of five animals, in which tumor was induced, remained untreated. Mice were then euthanized 3 days after; tumors were collected, processed for histological and iron content analysis (Prussian blue staining). The % of blue Prussian staining and standard area from 5 randomly chosen areas from each of the 3 tumor sections (100 microns apart) for each of the 7 tumors (n = 105) were analyzed with ImageJ software.

### 2.10. Statistical Analysis

Data are expressed as mean ± standard error of at least 3 triplicates. Statistical analyses were performed using a one-way ANOVA with Bonferroni’s post-test for grouped analyses using GraphPad Prism version 4.03 for Windows, GraphPad Software (GraphPad Prism, San Diego, CA, USA). One representative experiment out of the three performed is shown, except for the experiments carried out in vivo. Statistical analysis of the presence of nanoparticles in tumor sections was performed using unpaired t test and F test for unequal variance on the measures obtained. The results are shown as box and whisker plots, showing the minimum, first quartile, median, third quartile, and maximum measures. Differences at *p* < 0.05 were statistically significant. Statistical differences between the treatments were considered significant when p values were *p* < 0.05, *p* < 0.01, *p* < 0.001, labelled in the figures as *, **, ***, respectively.

## 3. Results and Discussion

### 3.1. Cytocompatibility of Magnetic Nanoparticles

**Figure 2:**
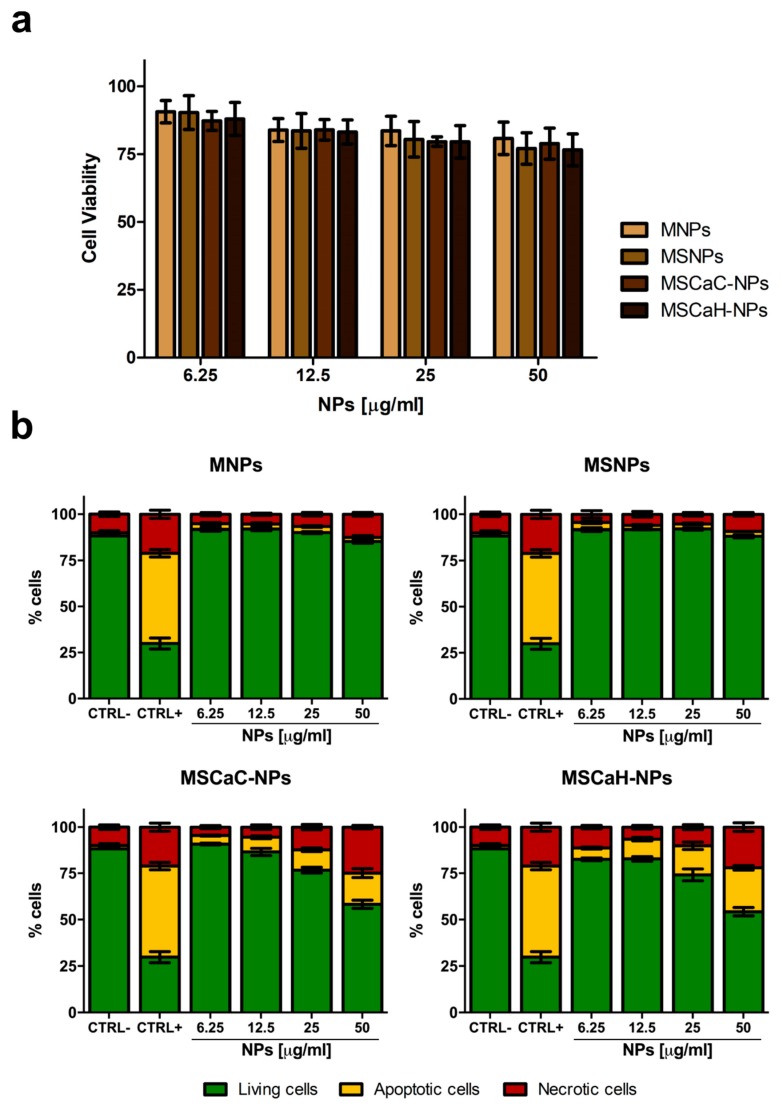
Cytocompatibility of magnetic nanoparticles. Viability of GTL-16 cells was assessed after three days incubation in MTT assay (**a**) or in an annexin V-propidium assay (**b**). In (**a**) data are expressed as % cell viability with respect to control untreated cells and no statistically significant differences were observed. In (**b**) CTRL− refers to control untreated cells and CTRL+ refers to cells incubated with 400 mM hydrogen peroxide. For statistical analysis all data were compared vs. untreated samples. Significant toxicity was observed only for treatments of 25 and 50 µg/mL calcium citrate (MSCaC-NP) and calcium hydroxide (MSCaH-NP).

The cytotoxicity of nanocarriers is a key factor for their eventual potential biomedical applications. Therefore, the four types of NPs, already characterized and used in different systems [38,39], were tested for their cytocompatibility on the GTL-16 human gastric carcinoma cell line, in view of their further use in in vivo experiments after functionalization with a targeting mAb. Cells were incubated with increasing doses of MNPs (from 6.25 µg/mL to 50 µg/mL) for 72 h and analyzed by MTT assays. In these static exposure conditions, a slight reduction in cell viability was observed in a dose-dependent manner (Figure 2a), which, however, was always higher than 80%. Indeed, these values are above the cytocompatibility cut-off (70%) indicated by ISO 10993-5:2009 [48]. The same results were observed when the L929 normal fibroblast cell line was incubated with the four types of MNPs (not shown). Cytocompatibility was also assessed in an annexin V/propidium iodide test, which was able distinguish between apoptotic and necrotic cells. In this case, pure MNPs, as well as MNPs covered with a Silica shell (MSNPs), behaved as in the MTT assay, while both types on MNPs doped with calcium (MSCaC-NP and MSCaH-NP) induced a significant level of apoptosis (ca. 15% and 20%) and necrosis (22% and 20%), when used at the highest concentrations (Figure 2b). Different results were observed for calcium-doped MNPs which can be accounted for by the fact that the two assays measured different parameters, the former an enzymatic activity of the mitochondrion, the latter a biochemical modification of the external cell surface occurring precociously in apoptosis [49]. The detrimental effect exerted by calcium can be ascribed to the fact that the level of this ion controls many biological activities, and thus must be finely tuned both in the extracellular and in the intracellular environments [50]. An uncontrolled increase in its levels might be cytotoxic, by inducing oxidative stress, and thus promoting cellular apoptosis and necrosis [51]. Thus, on the basis of these cytocompatibility assays, the two best performing nanoparticles (MNPs and MSNPs) were selected for further experiments of functionalization, discarding the two MNPs containing calcium.

### 3.2. Functionalization of Magnetic Nanoparticles

**Figure 3:**
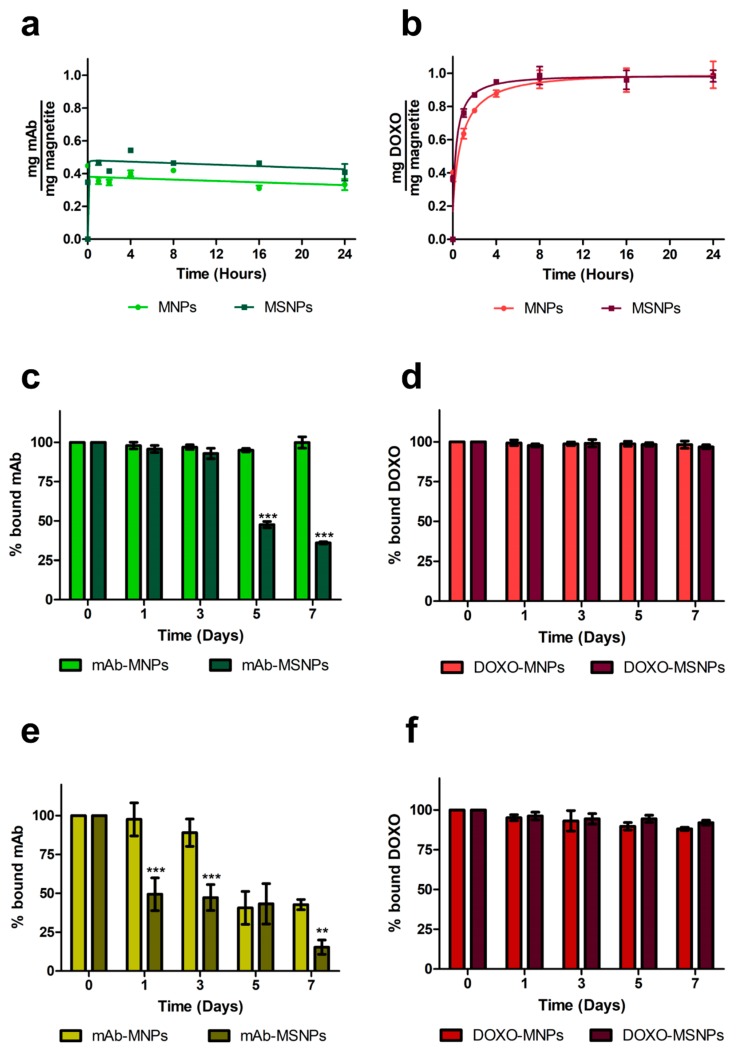
Functionalization of magnetic nanoparticles and stability of the complexes. Nanoparticles were incubated for different times under constant stirring (200 rpm) at 37 °C with DO-24 monoclonal antibodies (mAbs) (**a**) or doxorubicin (DOXO) (**b**) for isothermal adsorption. The washed complexes (mAb-NPs, **c** and **e** and DOXO-MNPs, **d** and **f**) were then incubated for different times under constant stirring (200 rpm) at 37 °C in PBS not containing (**c**,**d**) or containing (**e**,**f**) 10% FCS. The particulate and the soluble fractions were separated through a magnet and mAb bound on nanoparticles was estimated in SDS-PAGE and WB (A), while DOXO bound was calculated by subtracting the amount detected in the soluble fraction (B). For statistical analysis the treatments were compared between each other at each time point (*** *p* < 0.001).

Functionalization was carried out with the DO-24 mAb, which recognizes the ectodomain of the human Met/HGFR, which is overexpressed at the surface of many tumors [32,52] and with DOXO, which is one of the most used chemotherapeutic drugs. The two molecules were bound via isothermal adsorption, to better preserve the biological activity of the mAb. Coupling of the mAb to both nanoparticle types was efficient since the first incubation time assessed, with values of about 35% and 46% of the initially incubated mAbs to MNPs and MSNPs, respectively (Figure 3a), resulting in 70 to 92 µg mAb bound/mg NP. In the case of DOXO, the maximum binding was reached with a certain delay (after 4 h incubation for MSNPs and 8 h incubation for MNPs) and no significant differences in the bound amounts (96% ± 0.05 in the case of MNPs and 99% ± 0.06 in the case of MSNPs, corresponding to nearly 200 µg Doxo bound/mg NP) were observed (Figure 3b). This very high percentage can be explained based on the properties of the DOXO molecule, which carries both negative and positive charges and has the propensity to form dimers in solution [53] and can potentially be adsorbed on multiple layers on NPs. This would also explain the observed delay in reaching the plateau. The fact that a lower percentage of the mAb bound to the MNP surface can also be ascribed to the steric hindrance it exerts, because it has a molecular mass approximately 275-fold than that of DOXO.

### 3.3. Characterization of Magnetic Nanoparticles

The stability of the different complexes, in which the functionalizing moieties are bound by electrostatic interactions, was then analyzed by evaluating the amount of the two ligands remaining bound to nanoparticles after incubation in PBS at 37 °C in continuous stirring for different times up to seven days. The two immunocomplexes behaved similarly up to three days incubation with no release of mAb (Figure 3c). However, after this period more than 50% of the adsorbed mAb was released from MSNPs, while none from MNPs. In the presence of 10% FCS, mAbs were released more precociously from both MNPs types (Figure 3e) and also, in this case, MNPs performed better than MSNPs, because only 10% of the adsorbed mAb was released from the former after three days vs. 50% from the latter after only one day. For longer times, mAb release from both NP types increased, but at lower levels in the case of MNPs. The stability of mAb-MNPs for three days in the presence of 10% FCS should be a time long enough to allow them to reach their targeted molecules on tumor cells. The stability of the immunocarrier could be improved by covalent binding of the mAb, but this would inevitably involve more complex synthetic procedures and the introduction of some chemical reagents with potential toxicity problems [54]. It was also reported for one case in which DOXO was therapeutically active when it was noncovalently entrapped in the nanoconstruct, but not when it was coupled via covalent amide bonds [55].

In the case of DOXO-functionalized nanoparticles, less than 2% to 2.5% of the bound chemotherapeutic was desorbed from both types of NPs in PBS, and less than 10% was released after incubation in the presence of FCS, suggesting highly stable complexes in all cases (Figure 3d,f). When DOXO was coupled to mAb-functionalized MNPs, about 40% of the input amount was adsorbed and also, in this case, only 2% to 2.5% of the bound DOXO was released (not shown).

**Figure 4:**
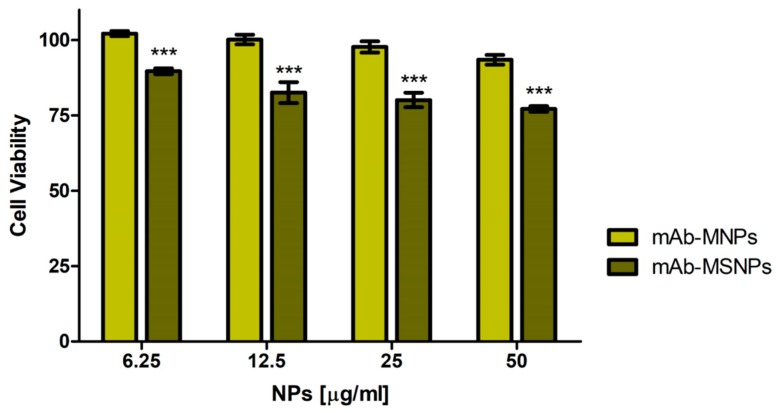
Cytocompatibility of the mAb-functionalized magnetic nanoparticles. Viability of GTL-16 cells was assessed in MTT assays after three days incubation. Data are expressed as % cell viability with respect to control untreated cells. For statistical analysis all data were compared vs. untreated samples (*** *p* < 0.001).

The two immunocomplexes, obtained by coupling of MNPs and MSNPs, respectively, were then tested for their cytocompatibility on GTL-16 in an MTT assay performed after three days incubation. The mAb-MNPs were highly cytocompatible, because more than 95% of the cells were fully viable at the highest dose used of 50 µg/mL, whereas, in the case of mAb-MSNPs, cell viability was somehow decreased (lower than 80%), in line with the results and values observed for not functionalized MSNPs (Figure 4). Thus, also from this point of view, MNPs displayed better properties.

**Figure 5:**
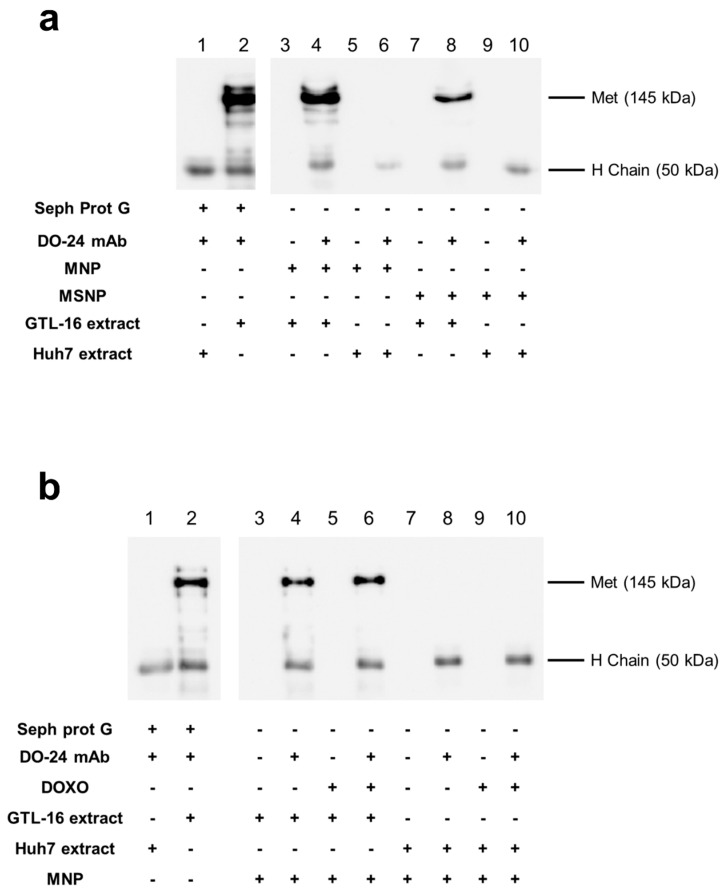
Immunocompetence of the functionalized magnetic nanoparticles. Nanoparticles differentially functionalized were incubated with detergent extracts from GTL-16 (Met+) or Huh7 (Met−) cells; bound proteins were analyzed in SDS-PAGE and WB with anti-Met antibodies and secondary peroxidase-labelled anti-mouse IgG antibodies. Only, when the mAb and GTL-16 extracts are incubated together, could a band with 145 kDa molecular weight be detected, corresponding to the Met proteins, lanes 2, 4, and 8 in panel (**a**) and lanes 2, 4, and 6 in panel (**b**). When mAb was present in the incubated mixtures, a band 50 kDa, corresponding to the mAb heavy chain, was detectable (even lanes from #3).

The ability of the mAb-functionalized magnetic NPs to react with the cognate antigen, i.e., their immunocompetence, was assessed in immunoprecipitation experiments with extracts from cells expressing the Met/HGFR antigen or not. Only the MNPs coupled with the mAb, but not the MNPs devoid of the mAb, were able to precipitate a 145 kDa band, corresponding to the Met/HGFR β chain, from samples obtained from the Met/HGFR+ GTL-16 cells (Figure 5a, lanes 4 and 8 vs. lanes 3 and 7). The capability to immunoprecipitate Met/HGFR is not the same among the functionalized MNPs, since mAb-MNPs appeared to display a better activity than mAb-MSNPs in terms of interaction with the antigen. The 145 kDa band could not be detected in the immunoprecipitation performed with mAb-coupled MNPs reacted with extracts from Huh7 cells, which do not express Met/HGFR. In these cases, only the 55 kDa band, corresponding to the mAb heavy chain, were visualized (Figure 5a, lanes 6 and 10) and, as expected, no bands were detected when the precipitation was performed with MNPs devoid of mAbs (Figure 5a, lanes 5 and 9). All together these data support the specificity of the mAb-MNPs. The same experiment was performed also incubating ternary MNPs with GTL-16 cell extracts. In this case, no differences were detected in the ability of mAb functionalized MNPs either carrying DOXO or not (Figure 5b, lanes 4 and 6 vs. lanes 3 and 5), showing that DOXO adsorption did not impair the immunocompetence of mAb-loaded MNPs. Moreover, these data suggest that, in all the cases, the Fab domains of the mAb should be exposed externally, possibly in an end-side orientation imposed also by the steric hindrance of the Fabs relative to the Fc domain, in agreement with the theoretical work of Sidorov [56] and similar published data [45,57]. In both series of these experiments, the Met/HGFR was precipitated also by mAb adsorbed on Sepharose Protein G, which is the gold standard condition for immunoprecipitations (Figure 5a,b, lane 2 vs. lane 1) and no bands were detected when not functionalized nanoparticles were used.

**Figure 6:**
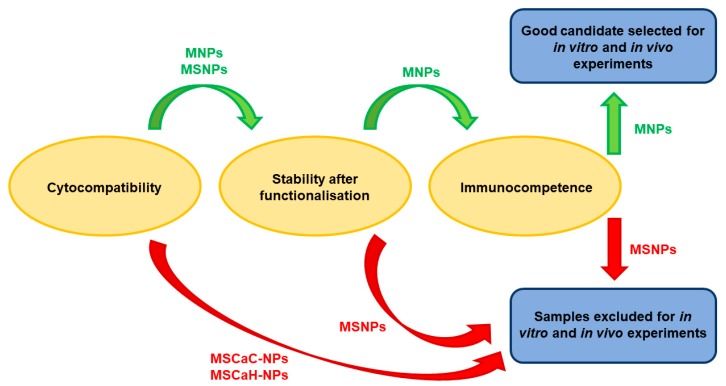
Selection of the best performant nanoparticle.

After all these experiments, including cytocompatibility, stability, and immunocompetence, MNPs appeared to be the best performants, and thus they were selected for further studies (Figure 6).

MNP and mAb-MNPs underwent further characterization. They were characterized for their sizes with DLS measurements in PBS. The usual size of these MNPs is around 10 nm to 13 nm, showing the tendency to form agglomerates of different sizes (Table 1), as already reported in the literature for this type of compounds [58,59]. It is worth noting that most of the agglomerates of bare MNPs (97%) are of size fully compatible with the interaction with cells. Further functionalization of MNPs with mAb and DOXO slightly perturbed the agglomerates distribution, even if smaller agglomerates remain predominant. The ζ potential value determined for MNPs (−18.55 ± 0.86 mV) is in accordance with literature data [60,61]. The functionalization impacted on the ζ potentials, which turn positive after DOXO and Mab coupling [62].

**Figure 7:**
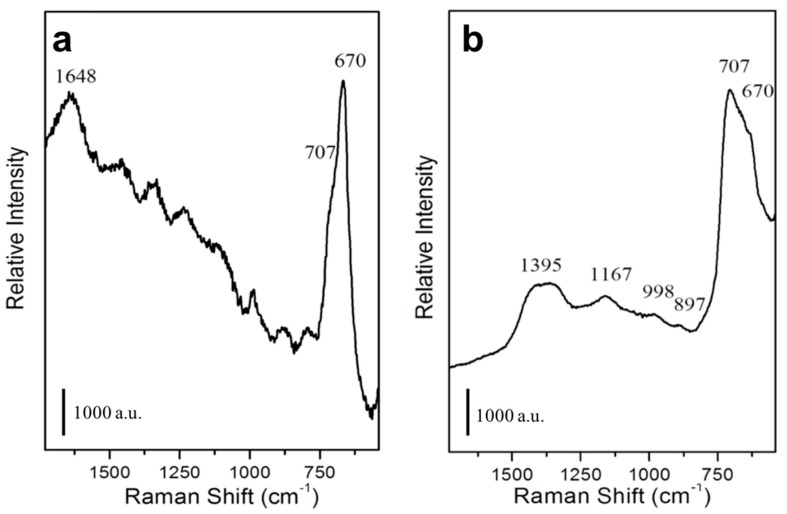
Characterization of mAb-functionalized magnetic nanoparticles. Raman spectra of not functionalized (**a**) or mAb-functionalized (**b**) MNPs.

The Raman spectrum of the not functionalized MNPs is characterized by a signal at 670 to 707 cm^−1^ (Figure 7a), typical of Fe_3_O_4_ [63]_._ An additional intense signal at 1648 cm^−1^ is ascribable to HOH bending mode of water molecule, whereas other minor signals are present due to stretching modes of phosphate species [64] of the PBS buffer. In the Raman spectrum of mAb functionalized MNPs (Figure 7b), in addition to the signal of Fe_3_O_4_ at 670 to 707 cm^−1^, signals in the 1000–1500 cm^−1^ range are also present and they are ascribable to the amide groups of the mAb [45].

### 3.4. Interaction of mAb Functionalized Magnetic Nanoparticles with Cells

**Figure 8:**
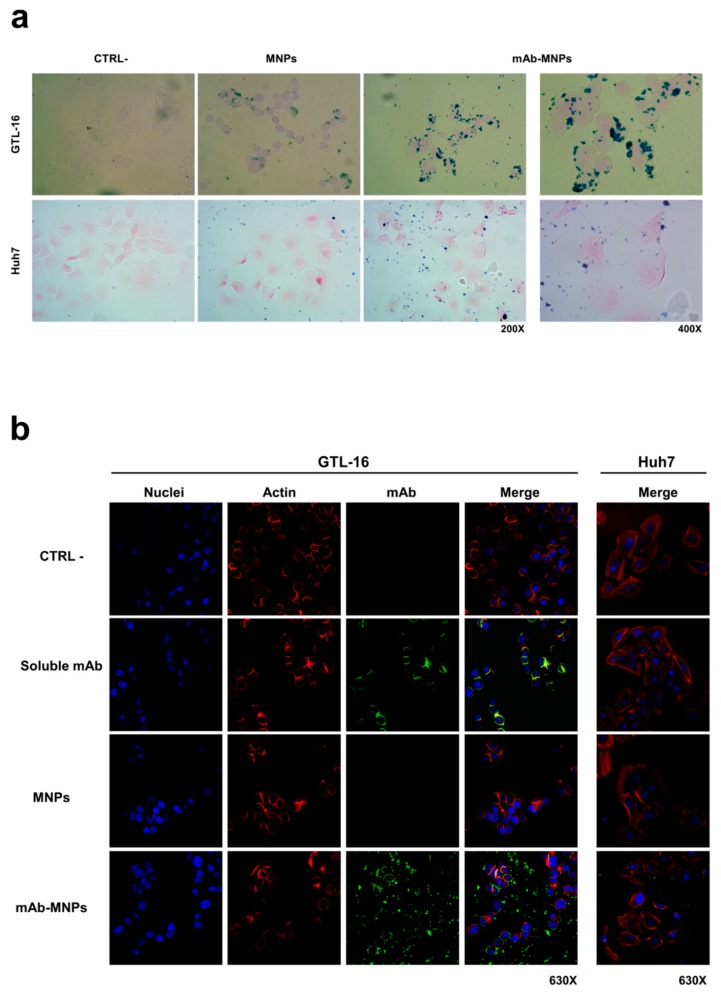
Interaction of mAb-functionalized magnetic nanoparticles with cells. GTL-16 and Huh7 cells were incubated with MNPs, functionalized or not with the DO-24 mAb (**a**) and (**b**), as well as with soluble mAb (**b**) for 2 h at 37 °C. After washing, staining was performed with Prussian blue (**a**); the first three panels on the left are 200× magnification and the forth on the right is 400× magnification for both cell types) or with FITC-labelled anti-mouse Ig (green), TRITC-labelled falloidin (red), TO-PRO3 (blue) to label the DO-24 mAb, the cytoskeletal actin and nuclei, respectively (**b**). Images were then merged. CTRL−, control untreated cells.

The interaction of these MNPs with cells after incubation at 37 °C for 2 h was evaluated by Prussian blue staining, which tracks magnetic nanoparticles as blue dots. The mAb-MNPs were clearly detectable at the surface of Met/HGFR+ GTL-16 cells, due to the presence of the DO-24 mAb, whereas MNPs devoid of the mAb were visible at significantly lower levels (Figure 8a, top row). The same low level of detectability was also found when the mAb-functionalized or not functionalized MNPs were incubated with the Met/HGFR- Huh7 cells, where also a more random distribution of the few MNPs was visualized (Figure 8a, bottom row). These data show that the DO-24 mAb coupled MNPs are able to specifically interact with the cells expressing the Met/HGFR tumor marker; in these conditions these functionalized MNPs do not appear to be able to be internalized within cells, most probably because they form agglomerates.

The interaction of the mAb-MNPs with cells was evaluated by also tracking the mAbs in indirect immunofluorescence experiments examined in confocal laser scanning microscopy (Figure 8b). The signal due to the FITC-conjugated secondary antibody was evident only when mAb-functionalized MNPs were incubated with GTL-16 cells (Figure 8b, fourth row). As in the case of the experiments performed with Prussian blue staining, also in this case, MNPs, which were made detectable in indirect way, were present at the cell surface, but they were not internalized within cells. By contrast, in samples where soluble mAbs were incubated with GTL-16 cells, mAbs were found within the cells, since a yellow signal was detectable, resulting from the superimposition of the two signals, the green corresponding to the mAb and the red to the cell cytoskeleton (Figure 8b, second row). Finally, no signal was detectable when this kind of experiment was performed on Huh7 cells (Figure 8b, right column) or on both cell targets with not functionalized MNPs (Figure 8b, third row). Thus, these data agree with and confirm the results obtained with Prussian blue staining. They show a mAb-dependent and specific interaction of the MNPs with cells targeted by the mAb. In this context, additionally, it should be considered that in vivo the mAb-functionalized MNPs would bind preferentially to cells expressing higher amounts of the recognized marker, such as tumor cells, among all the cells they encounter [45].

**Figure 9:**
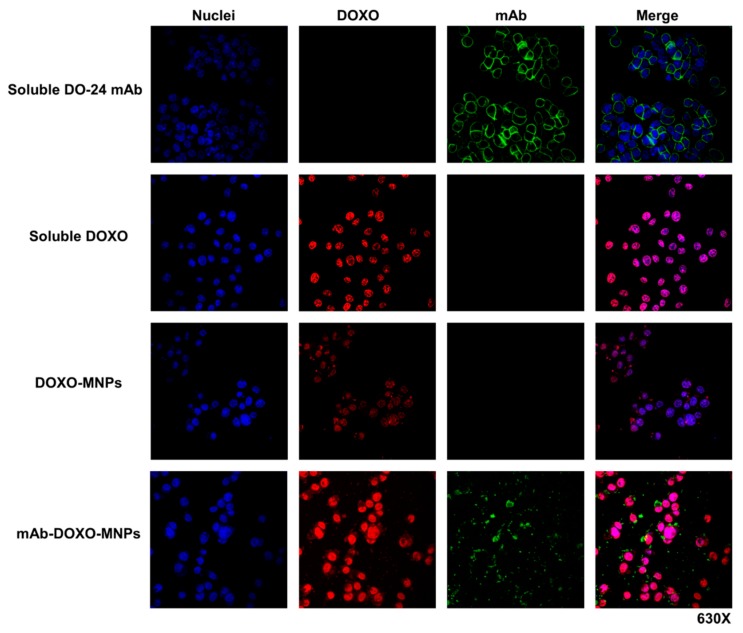
Interaction of mAb-DOXO-functionalized nanoparticles with GTL-16 cells. Cells were incubated with mAb-functionalized MNPs, carrying or not DOXO, as well as with soluble DOXO for 2 h at 37 °C. After washing, staining was performed with FITC-labelled anti-mouse Ig (green), TO-PRO3 (blue) to label the DO-24 mAb and nuclei, respectively. DOXO is detected as a red signal. Images were then merged.

In another set of experiments ternary MNPs functionalized with both mAbs and DOXO were incubated with GTL-16 cells for 2 h. In this case, too, mAbs were detected at the surface of the cells labelled in green by FITC-coupled secondary antibodies, but the red fluorescent DOXO was found in the cell nuclei, strongly suggesting that it was discharged from MNPs, internalized and able to reach nuclei, where it could eventually exert its toxic activity (Figure 9, fourth row). In the same series of experiments, when cells were incubated either with soluble DOXO (Figure 9, second row) or with DOXO-functionalized MNPs (Figure 9, third row), this drug was detectable in the nuclei, but at clearly lower levels in the latter case. It is thus shown that the presence of mAb on DOXO-functionalized MNPs enhances the cellular uptake of the drug. While DOXO is discharged intracellularly, the mAb was found only extracellularly associated to the cells surface, possibly because the former was present in a more external position on the nanoparticle as compared with DO-24 mAb which was adsorbed first. The fact that in vivo tumor microenvironment is generally acidic (pH < 6) would favor the dissociation of DOXO from MNPs. Indeed, at these pH values the strength of the electrostatic bonds between the particle and the adsorbed molecules should be decreased, facilitating desorption. In these conditions it cannot be excluded that a certain amount of mAb could also be released from MNPs in vivo in the tumor cells environment, however, once in this site, the mAbs should have already performed their targeting activity.

### 3.5. Enhanced Cytotoxicity of mAb-Functionalized DOXO-Loaded MNPs on Specifically Targeted Cells

**Figure 10:**
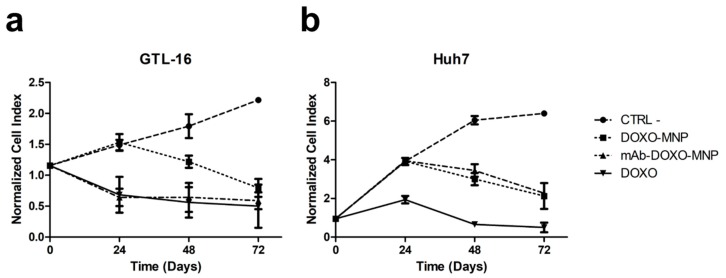
Cytotoxicity of mAb-DOXO-functionalized nanoparticles. Real-time toxicity of the not functionalized MNPs, of the MNPs functionalized only with DOXO, or only the mAb or with both DOXO and mAb were performed on the Met/HGF-R+ GTL-16 and on the Met/HGF-R- Huh7 cells. The presence of the DO-24 mAbs significantly increase the toxic activity of the DOXO-functionalized MNPs on GTL-16 cells (**a**), whereas it had no effect on Huh7 cells (**b**). Cell index, indicative of cell viability, was measured in a xCELLigence^®^ assay. CTRL−, control untreated cells. Incubation with not functionalized MNPs gave results similar to those of the control untreated cells.

Next, the toxicities exerted by the binary MNPs functionalized with DOXO and that of the ternary MNPs also carrying the targeting mAb were compared in an xCELLigence^®^ assay performed on GTL-16 cells. A significant difference (*p* < 0.001) was observed, because the presence of the mAb on MNPs strongly increased the toxicity on GTL-16 cells in respect to that displayed by binary MNPs both at 24 and 48 h (Figure 10a). Indeed, in the former case, the level of toxicity is superimposable to that of soluble DOXO, while that of binary DOXO-MNPs is significant lower, in line with what already has been reported for other nanoparticles [45,57]. In parallel control experiments performed on Huh7, which do not express the relevant marker, no differences in the toxic activities of the ternary versus the binary MNPs were observed and, in any case their toxicity was lower than that of soluble DOXO (Figure 10b). Thus, MNPs allow decreasing the aspecific DOXO cytotoxicity and, if functionalized with the mAbs, allow focusing it on targeted cells, sparing cells, which do not express Met/HGFR.

All together these data show the positive effect and the specific activity of the mAb coupled on MNPs on cells expressing the corresponding targeted receptor. NPs were shown to enhance the anti-tumor activity of the bound anti-Met nanobodies also with another mechanism. In this case, crosslinked albumin NPs decorated with these nanobodies were better uptaken by cells and were more efficient than soluble nanobodies in downregulating the Met receptor and inhibiting cell motility [65].

### 3.6. In Vivo Distribution of Bare MNPs

**Figure 11:**
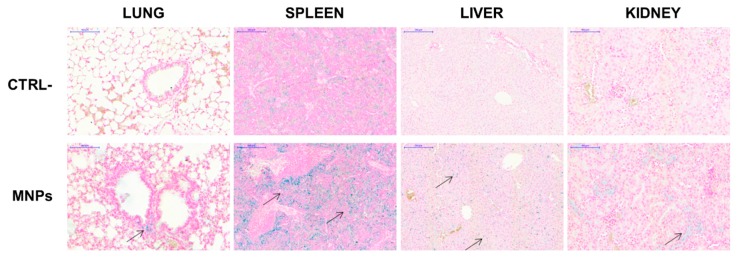
In vivo distribution of MNPs. NOD/SCID-γ^null^ mice were injected in the tail vein with MNPs (10 µg/g) in 100 µL PBS. After 3 days these mice, together with untreated control mice, were euthanized and their organs were recovered and processed for histological analysis after staining with Prussian blue. Arrows indicate iron deposits stained in blue.

Before in vivo experiments with tumors-bearing mice, the biocompatibility and the distribution of not functionalized MNPs was determined in NOD/SCID-γ^null^ mice after systemic administration by tail vein injection. The dose (10 µg/g mouse) to be injected for these in vivo experiments was chosen according to studies previously published with other MNPs [66,67] and previous experience with these same MNPs in a different mouse strain [38]. No distress signals were observed at day three, as well as after one month, the latest time point analyzed. No obvious organ damages or toxic side effects were observed after Prussian blue staining of the histological sections performed at day three (Figure 11). At the dose of MNPs used, only spleens showed significant blue deposits, whereas lung, liver, and kidney were stained at significantly lower levels, suggesting minimal deposits of MNPs. No iron signal could be detected in the heart and in the brain (data not shown). These data demonstrate that, even if the MNPs have the tendency to form agglomerates, they do not display acute toxicity.

### 3.7. Intratumoral Retention of mAb-MNPs Complexes after In Situ Injection

**Figure 12:**
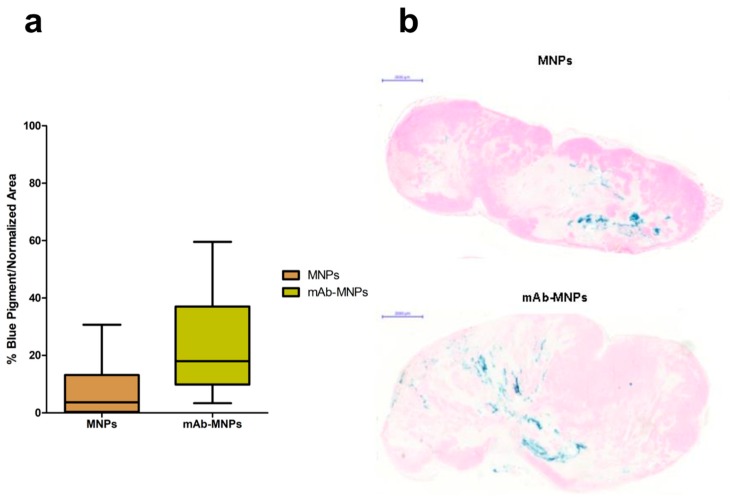
Distribution of mAb-targeted and not targeted MNPs in Met oncogene positive (Met/HGFR+) GTL-16 cells induced tumors. NOD/SCID-γ^null^ mice bearing GTL-16 cells induced tumors were injected intratumorally with MNPs functionalized or not with the DO-24 mAb and killed after 3 days. Tumors were recovered and processed for staining with Prussian blue. Box and whisker plots summarize the results obtained from measuring the distribution and quantification of MNPs in the tumors (**a**). The % of Prussian blue staining and standard area from 5 randomly chosen areas from each of the 3 tumor sections (100 microns apart) for each of the 7 tumors (n = 105) was determined. Each plot shows the minimum, first quartile, median, third quartile, and maximum values of these % for tumors treated with MNPs and mAb-MNPs. Statistical analyses showed significant differences between the treatments, with a *p* < 0.0001 and *p* < 0.0008 for *t* and F tests, respectively. Representative tumor sections from mice receiving not functionalized or mAb- functionalized MNPs (**b**).

The ability of the mAbs coupled on MNPs to direct their interaction with cells expressing the Met/HGFR marker was tested in vivo in NOD/SCID-γ^null^ mice bearing tumors induced by subcutaneously injected human GTL-16 cells and grown until clearly detectable (~0.5 cm Ø, around day seven to 10). At this time, mice were intratumorally injected with MNPs, either functionalized with the mAb or not functionalized as the control. MNPs were delivered intratumorally, since in previous experiments performed with these MNPs coupled with lentiviral vectors, these complexes could not reach the tumor after MNPs iv injection, even when a magnet was superimposed at the tumor site [38]. This result could be explained by the observation that generally tumors have highly heterogeneous blood vessel distributions and strong stromal barriers to drug delivery systems [68]. Mice were killed three days after nanoparticles administration. In both cases, MNPs could be visualized by Prussian blue staining. Some differences in their distribution patterns were detectable. In the case of mAb functionalized MNPs, these were detected at higher levels and more spread among the tumor mass, while in the case of not functionalized MNPs, these showed the tendency to remain closer to the site of injection (Figure 12). These data indicate that the presence of the mAbs on MNPs allowed them to reach a higher number of tumor cells and to be retained there. An in vivo tumor targeting effect of the mAb coupled to MNPs can thus be evinced from these experiments. In addition, in the case of the MNPs coupled with lentiviral vectors, a targeting effect was observed if NPs were injected intratumorally and a continuous magnetic field was applied, because more of these functionalized MNPs were retained there in respect to untreated tumors [38].

These data demonstrate that, in the future, dual targeting could be exploited. In this way the combination of mAb-dependent chemical targeting and of physical magnetic targeting should enhance preferential accumulation at the target site [18,69]. Moreover, also at therapeutic level, the tumor could be attacked by a combined strategy, i.e., chemotherapy and application of an alternating magnetic field producing heat, to which tumor cells are more sensitive than normal cells (thermoablation), and which in the meantime enhances drug release from MNPs [15,16,17,70].

## 4. Conclusions

The possibility to guide therapeutic molecules on tumor cells by active targeting cell membrane tumor markers, allowing to decrease the systemic side effects of the drug, is becoming a reality thanks to multifunctional nanocarrier platforms. In this study, we coupled magnetic nanoparticles with DOXO and a monoclonal antibody recognizing the Met/HGFR which is overexpressed on several tumor types and can thus be considered a tumor-associated marker. Among the different MNPs analyzed, we found that the simplest ones, made of pure Fe_3_O_4_ were the most suitable for our experiments, being the most cytocompatible, the most stable after functionalization, and the best for Met/HGFR reactivity. The mAb-functionalized MNPs interacted specifically with the surface of cells expressing Met/HGFR, they were not internalized, but, when they were also coupled with DOXO, they released DOXO within the cells and they were more efficient in killing these cells as compared with MNPs coupled only with DOXO. The aspecific drug cytotoxicity was significantly decreased upon its coupling on MNPs, as shown on Met/HGFR negative cells.

Finally, when injected in vivo in tumors expressing Met/HGFR, mAb-coupled MNPs were able to target more efficiently than bare MNPs tumor cells, since they were better dispersed within the tumor mass. Thus, these MNPs should be considered new and promising nanocarriers for targeted drug delivery opening new perspectives for in vivo studies, in which continuous and alternating magnetic fields could be applied to enhance targeting and to induce hyperthermia, respectively.

## Figures and Tables

**Table 1:** Properties of magnetic nanoparticles (MNPs).

Acronym	Particle Size * (%)	Zeta Potential (mV)
MNPs	101 ± 15 (97%) 1229 ± 288 (3%)	−18.55 ± 0.86
mAb-MNPs	135 ± 19 (86%) 815 ± 200 (14%)	9.26 ± 0.93
DOXO-MNPs	145 ± 21 (78%) 790 ± 180 (22%)	5.4 ± 0.24
mAb-DOXO-MNPs	132 ± 35 (84%) 654 ± 82 (16%)	8.8 ± 0.55

* nm.
